# Potential Value of Cerebrospinal Fluid Progranulin in the Identification of Postoperative Delirium in Geriatrics Patients Undergoing Knee Replacement: The Perioperative Neurocognitive Disorder and Biomarker LifestylE Study

**DOI:** 10.3389/fnagi.2021.772795

**Published:** 2022-01-06

**Authors:** Bin Wang, Xiujie Sun, Jiahan Wang, Xiyuan Deng, Yanan Lin, Fanghao Liu, Rui Dong, Xu Lin, Yanlin Bi

**Affiliations:** ^1^Department of Anesthesiology, Qingdao Municipal Hospital Affiliated to Qingdao University, Qingdao, China; ^2^Department of Nursing, Qingdao Municipal Hospital Affiliated to Qingdao University, Qingdao, China; ^3^Department of Anesthesiology, Dalian Medical University, Dalian, China; ^4^Department of Anesthesiology, Weifang Medical University, Weifang, China; ^5^Department of Anesthesiology, Drum Tower Hospital Affiliated to Nanjing University Medical School, Nanjing, China

**Keywords:** progranulin, biomarkers, neurodegeneration, geriatric, postoperative delirium (POD)

## Abstract

**Objective:** The aim of this study was to investigate whether progranulin (PGRN) levels in cerebrospinal fluid (CSF) were associated with postoperative delirium (POD) in geriatric patients undergoing knee replacement.

**Method:** A total of 600 Han Chinese patients aged 65–90 years and who underwent unilateral total knee arthroplasty were included in the Perioperative Neurocognitive Disorder And Biomarker LifestylE (PNDABLE) study from June 2020 to November 2020. All participants were assessed using the Confusion Assessment Method and the Memorial Delirium Assessment Scale on postoperative days 1–7 (or before discharge) by an anesthesiologist. CSF PGRN and CSF biomarkers of POD were measured by ELISA. We analyzed the risk and protective factors of POD using the multivariate logistic regression, and the associations between CSF PGRN and CSF biomarkers of POD using multiple linear regression. We also explored whether the influence of CSF PGRN on POD was mediated by POD core pathology in linear regression models.

**Results:** Postoperative delirium incidence was 9.7% (53/545). There were significant differences in preoperative CSF PGRN between patients with POD and non-POD (NPOD). As for CSF biomarkers, CSF Aβ_40_, T-tau, and P-tau were risk factors for POD, while CSF PGRN, Aβ_42_, and Aβ_42_/Aβ_40_ were protective factors for POD, as shown by the multivariate logistic regression analysis. CSF PGRN was positively associated with CSF Aβ_42_ and was negatively associated with CSF Aβ_40_, T-tau, and P-tau in patients with POD. We found that the AUC was 0.795 (95% CI = 0.706, 0.867) for PGRN between POD and NPOD groups. We found the influence of CSF PGRN on POD was mediated by POD core pathology. The effect was considered partial mediation with the proportion of mediation varying from 44.92 to 62.07%.

**Conclusion:** Cerebrospinal fluid PGRN may be a reasonably good prognostic factor for POD development. Overall, amyloid pathology and tau protein might partially mediate the influence of PGRN on POD.

**Clinical Trial Registration:** www.clinicaltrials.gov, identifier ChiCTR2000033439.

## Introduction

Postoperative delirium (POD) represents a serious complication following anesthesia and surgical procedures for patients undergoing surgical intervention (Safavynia and Goldstein, 2018). Postoperative delirium is characterized by temporary or permanent cognitive decline, as well as deterioration in language comprehension and social adaptation abilities, and POD particularly affects geriatric people (> 65 years) (Ramaiah and Lam, 2009). Postoperative delirium can lead to increased mortality, prolonged hospitalization, other complications such as Alzheimer’s disease (AD), and higher treatment costs (Steinmetz et al., 2009). Despite the prevalence and clinical importance of POD, its mechanisms are still poorly understood and no reliable biomarkers have been reported in previous studies.

Progranulin (PGRN), a multifunctional secretory protein, is a neurotrophic growth factor. The precursor proteins are hydrolyzed by extracellular proteases into smaller peptide fragments called GRNs or epithelins (Daniel et al., 2000). In the central nervous system, PGRN mainly exists in specific neurons, including microglial cells, cerebellar Purkinje cells, and hippocampal pyramidal neurons, which have functions including neurotrophy, axonal prolongation, promotion of neuron survival, and proliferation of neural stem cells (Philips et al., 2010). Studies have shown that PGRN in microglia cells may play an important role in brain injury, neuroinflammation, and neurodegeneration (Martens et al., 2012; Feng et al., 2020). Decreased PGRN expression in neurodegenerative diseases may be a self-protective mechanism to prevent cell damage in brain tissues. Some studies have found that PGRN protein is closely related to changes in cognitive function (Suárez-Calvet et al., 2018).

Aβ, including Aβ_40_ and Aβ_42_, is the main component of senile plaques in AD. Tau is a microtubule-associated protein in neurons, which is essential for microtubule formation and stability (Guo et al., 2017). Aβ and Tau have been considered as the biomarkers reflecting plaque pathology, neurodegeneration, and neurofibrillary tangle pathology for POD. A recent study has shown that preoperative positive cerebrospinal fluid (CSF) Aβ, T-tau, and P-tau may increase the risk for delirium following surgery (Fong et al., 2021). Other studies showed that preoperative positive CSF Aβ, T-tau, and P-tau were the strongest independent predictors of POD after elective arthroplasty in the elderly population without a prior diagnosis of dementia (Cunningham et al., 2019; Dutkiewicz et al., 2020). At present, it has been confirmed that β-amyloidosis and Tau phosphorylation are the two mechanisms underlying POD.

Progranulin protein was low when Aβ accumulation decreased, while PGRN could be upregulated by massive Aβ plaques (Minami et al., 2014). In a mouse model, tau hyperphosphorylation was significantly exacerbated due to reduced PGRN levels caused by mutations in GRN, demonstrating the role of PGRN in AD tauopathy (Hosokawa et al., 2015). Progranulin is associated with neurodegeneration in AD, and POD shares similar neuropathological mechanisms with AD. Therefore, we can infer that CSF PGRN may be associated with CSF Aβ, tau pathology, and neurodegeneration. Thus, the main objective of this study was to investigate whether CSF PGRN was a protective or risk factor for POD and whether the influence of CSF PGRN on POD was mediated by POD core pathology. These analyses were conducted based on the Perioperative Neurocognitive Disorder And Biomarker LifestylE (PNDABLE) study.

## Materials and Methods

### The Perioperative Neurocognitive Disorder and Biomarker LifestylE Study

The PNDABLE study intended to explore the pathogenesis, risk factors, and biomarkers of perioperative neurocognitive disorders in the northern Chinese Han population. Perioperative Neurocognitive Disorder And Biomarker LifestylE study aimed to identify lifestyle factors that may affect the risk of PND in the non-demented northern Chinese Han population to provide a basis for disease prevention and early diagnosis. This study has been registered in the Chinese Clinical Trial Registry (clinical registration number ChiCTR2000033439) and approved by the Ethics Committee of Qingdao Municipal Hospital. Cerebrospinal fluid samples were collected from all participants after written informed consent was obtained from the patients or their legal representatives.

### Participants

The Han Chinese patients undergoing unilateral total knee arthroplasty [no gender limitations, aged 65–90, weighted 50–80 kg, American Society of Anesthesiologist (ASA) I–II] combined with epidural anesthesia were enrolled in the PNDABLE study at Qingdao Municipal Hospital from June 2020 to November 2020. The exclusion criteria include (1) preoperative Mini-Mental State Examination (MMSE) score < 23 points; (2) drug or psychotropic substance abuse, as well as long-term use of steroid drugs and hormone drugs; (3) preoperative III–IV hepatic encephalopathy; (4) recent major surgery; (5) severe visual and hearing impairments; (6) abnormal coagulation function before surgery; (7) central nervous system infection, head trauma, multiple sclerosis, neurodegenerative diseases other than AD (e.g., epilepsy, Parkinson’s disease), or other major neurological disorders; (8) major psychological disorders; (9) severe systemic diseases (e.g., malignant tumors) that may affect CSF or blood levels of AD biomarkers including Aβ and tau; and (10) family history of genetic diseases.

**FIGURE 1:**
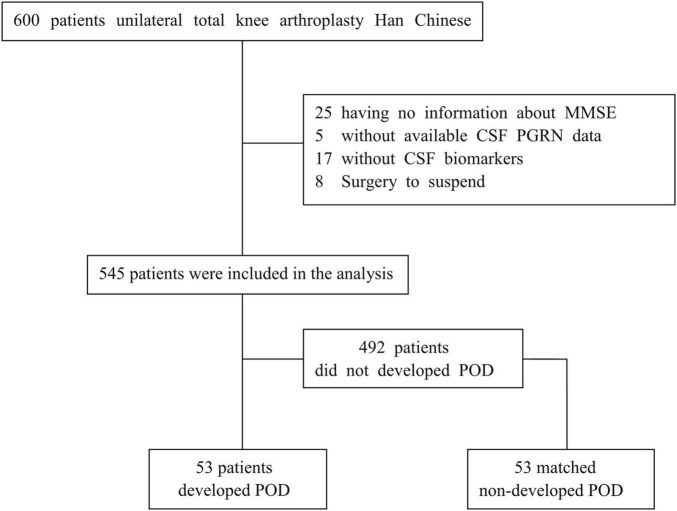
The flow diagram showed the selection of eligible patients and the enrollment process.

A total of 600 cognitively normal participants from the PNDABLE study had available information on covariates. According to the status of POD, participants were classified into POD and non-POD (NPOD) groups. Postoperative delirium cases and NPOD controls were frequency-matched (1:1) on age, the physical status of ASA, duration of surgery, and intraoperative blood loss. These variables were listed in the evidence- and consensus-based guidelines on POD from the European Society of Anesthesiology, and they were considered as risk factors for POD after knee replacement surgery. A patient recruitment flowchart is shown in Figure 1.

The participants did not receive preoperative medications, and they were instructed not to drink and eat for 6 and 8 h, respectively, before surgery. After entering the operating room, we routinely monitored ECG, SpO_2_, and NBP, opened vein access and extracted 3 ml of whole venous blood. All patients underwent a combined spinal-epidural block, with the space between lumbar 3–4 spinous processes (L3-4) as the puncture site. After the successful puncture, 2 ml of CSF was extracted from the subarachnoid space, followed by an injection of 2–2.5 ml ropivacaine (0.66%) for about 30 s. After administration of anesthesia, the sensory level was controlled below the T_8_ level. During the surgery, oxygen was inhaled *via* mask at 5 L/min to maintain blood pressure within ± 20% of the baseline value. If intraoperative NBP was <90 mmHg (1 mmHg = 0.133 kPa) or decreased by more than 20% of the baseline value, ephedrine of 5 mg was injected intravenously. If HR was <50 beats/min, atropine of 0.5 mg was injected intravenously. Intravenous patient-controlled analgesia (0.1 mg/ml butorphanol + 50 g/ml tropisetron, diluted with normal saline to a total volume of 100 ml) was used in acute postoperative pain management. After the operation, patients were sent to the post-anesthesia care unit (PACU).

We interviewed all the patients a day before surgery and collected their baseline data, including age, sex, body mass index (BMI), ASA physical status, and years of education. Other information including comorbidities, medical history, and fracture classifications were also collected according to the patients’ medical records. All the history collection, physical examination, and dementia-related cognitive assessment were conducted by an anesthesiologist.

### Cerebrospinal Fluid Biomarkers of Postoperative Delirium and Cerebrospinal Fluid Progranulin Measurements

The CSF samples were processed immediately within 2 h after standard lumbar puncture. Each sample was centrifuged at 2,000 × *g* for 10 min and then separated and stored in an enzyme-free Eppendorf (EP) tube (AXYGEN, Jiangsu, China; PCR-02-C) at −80°C under the international BIOMARKAPD project for further use in the subsequent steps of this study.

Cerebrospinal fluid PGRN and CSF biomarkers of POD were measured by ELISA on the microplate reader (X) (Thermo Scientific Multiskan MK3, Shanghai, China). CSF PGRN levels were measured using the specific ELISA kits (Human PGRN SimpleStep ELISA kit; No. RMEE103R; BioVendor, Ghent, Belgium) and CSF biomarkers of POD were measured using other ELISA kits [Aβ_40_ (BioVendor, Ghent, Belgium Lot: No.292-6230), Aβ_42_ (BioVendor, Ghent, Belgium Lot: No.296-64401), P-tau (BioVendor, Ghent, Belgium Lot No. QY-PF9092), and T-tau (BioVendor, Ghent, Belgium Lot No. EK-H12242)]. All ELISA measurements were performed by experienced technicians in strict accordance with the manufacturer’s instructions. They were blinded to the clinical information. The samples and standards were measured in duplicate, and the means of duplicates were used for the statistical analyses. All the antibodies and plates were from a single lot to exclude the between-batch variability. Moreover, the within-batch CV was < 5% and the inter-batch CV was <15%.

### APOE ε4 Gene Measurements

The DNA in the blood samples extracted using the QIAampDNA^®^ Blood Mini Kit (250) was separated and stored in an enzyme-free EP tube at −80°C until apolipoprotein E (APOE) genotyping in this study. Two specific loci related to APOE status (i.e., rs7412 and rs429358) were selected for genotyping with restriction fragment length polymorphism technology.

### Neuropsychological Tests

The preoperative cognitive status was assessed by neurologists using MMSE. Patients with an MMSE score <23 were excluded.

The assessment of delirium was performed at 9:00–10:00 a.m. and 2:00–3:00 p.m. two times a day on postoperative days 1–7 (or before discharge) by an anesthesiologist. We used the visual analog scale (VAS) score of 0–10 (lower scores indicating lower levels of pain) (Chung et al., 2016) to assess pain at the same time. POD was assessed by the Confusion Assessment Method (CAM) (Inouye et al., 1990), and POD severity was measured using the Memorial Delirium Assessment Scale (MDAS) (Schuurmans et al., 2003; Leung et al., 2008; Shi et al., 2014). Therefore, patients with CAM-positive and MDAS-positive on postoperative days 1–7 (or before discharge) were recorded. MDAS scores were recorded when POD occurred.

In the sixth month, cognitive function was assessed with the Telephone Interview for Cognitive Status-modified (TICS-m), a 12-item questionnaire that provides an assessment of global cognitive function by verbal communication *via* telephone; scores range from 0 to 50, with higher scores indicating better function. The quality of life was assessed by the World Health Organization Quality of Life brief version (WHOQOL-BREF), a 24-item questionnaire that provides an assessment of the quality of life in physical, psychological, social, and environmental domains. For each domain, the score ranges from 0 to 100, with higher scores indicating better function.

### Statistical Analysis

#### Sample Size Calculation

The preliminary test in this study found that 5 covariates were expected to enter the logistic regression. The POD incidence was 10%. And the loss of follow-up rate was assumed to be 20%. Therefore, the required sample size was calculated to be 600 (5 × 10÷0.1 × 1.2 = 600).

#### Outcome Analysis

The Kolmogorov–Smirnov test was used to determine whether the measurement data conformed to the normal distribution. Measurement data that conformed to the normal distribution were expressed as the mean e SD, while the median and interquartile range (IQR, 25–75 percentile) or a number (%) was used to express the data. The independent samples *t*-test was used for comparison, and the χ^2^ test was used for counting data between POD and NPOD groups.

First, the risk factors of POD were presented using a 95% CI. All selected risk factors and their associations with POD occurrence were examined using univariate logistic regression analyses. We also performed a multivariate analysis of factors related to POD using binary logistic regression adjusted for age, sex, years of education, and APOE ε4 carrier status.

Second, we also studied the associations between CSF PGRN and CSF biomarkers of POD (Aβ_42_, Aβ_40_, T-tau, and P-tau) in the multivariate linear regression adjusted for age, sex, years of education, and APOE ε4 carrier status. To examine whether the association between PGRN and POD was mediated by CSF POD biomarkers. The first equation regressed the mediators (CSF POD biomarkers) on the independent variable (PGRN). The second equation regressed the dependent variable (MDAS) on the independent variable. The third equation regressed the dependent variable on both the independent variable and the mediators. Mediation effects were established if the following criteria were simultaneously met: (1) PGRN was significantly related to CSF POD biomarkers; (2) PGRN was significantly related to MDAS; (3) CSF POD biomarkers were significantly related to MDAS; and (4) the association between PGRN and POD was attenuated or strengthened when CSF POD biomarkers (the mediators) were added in the regression model. Furthermore, the attenuation or indirect effect was estimated, with the significance determined using 10,000 bootstrapped iterations, where each path of the model was controlled for age, sex, years of education, and APOE ε4 carrier status.

Statistical significance was set at *P* < 0.05. SPSS statistical software, version 21.0 (SPSS, Inc., Chicago, IL, United States) and GraphPad Prism software, version 6.01 (GraphPad Software, Inc., La Jolla, CA, United States) were used for data analysis.

## Results

### Participant Characteristics

A total of 600 Han Chinese patients aged 65–90 years and who underwent unilateral total knee arthroplasty were included in the PNDABLE study from June 2020 to November 2020. We excluded 25 participants who had no information about MMSE, 5 participants without available CSF PGRN data, 17 participants who had no CSF biomarker data or had data outside four SDs of the mean, and 8 participants whose surgeries were suspended. Finally, 545 participants were included in this analysis, and they were divided into two groups (Figure 1). We found that the incidence of POD was 9.7% (*n* = 53 of the 545 patients) *via* our postoperative assessments. There were no significant differences between POD cases and NPOD controls in any of the four matched variables (i.e., age, ASA physical status, duration of surgery, and intraoperative blood loss), suggesting a successful matching procedure.

**FIGURE 2:**
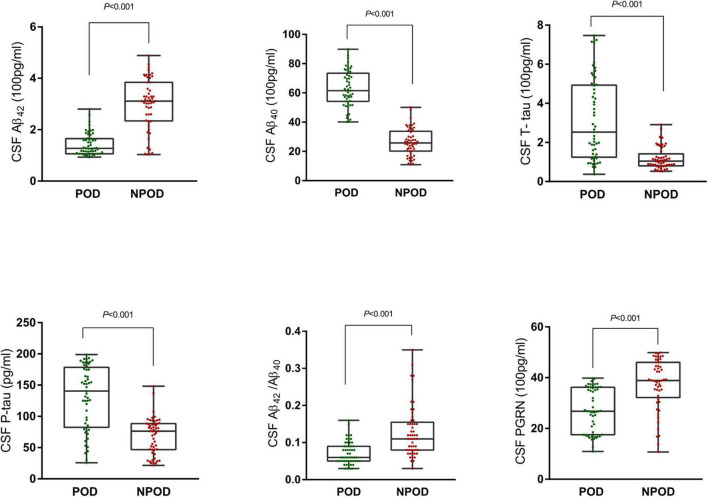
Distribution of cerebrospinal fluid (CSF) progranulin (PGRN) and biomarker levels for participants with and without delirium during subsequent hospitalization.

In this study, the differences in CSF levels of PGRN, Aβ_42_, Aβ_40_, Aβ_42_/Aβ_40_, T-tau, and P-tau were statistically significant between the POD group and the NPOD group (*P* < 0.05). We found that patients in the POD group had higher MDAS scores than the NPOD group. The preoperative MMSE score showed no significant difference between the POD group [28(26–29)] and the NPOD group [28(27–29), *P* = 0.330]. Postoperatively, the VAS score did not differ between patients with delirium 2(1–3) and those without delirium [2(1–3), *P* = 0.080]. We found that in patients of the POD group, cognitive function in the sixth month did not differ compared with the NPOD group. The demographic and clinical data of the participants were summarized (Figure 2 and Table 1).

**TABLE 1:** Comparison of general condition and surgical condition, CSF biomarkers of unilateral total knee arthroplasty patients.

Variable	POD(*N* = 53)	NPOD(*N* = 53)	P-values
Age (year), mean ± SD	72.85 ± 6.29	71.00 ± 5.73	0.117
Sex (female/male)	22/31	20/33	0.421
Body mass index (kg.m^–2^), mean ± SD	24.8 ± 3.6	25.7 ± 3.4	0.187
Education level (year), median and 25–75 percentile	9(6–13.5)	12(9–14)	0.326
ASA physical status (I/II)	27/26	28/25	0.846
APOE ε4 carriers (%), median and 25–75 percentile	7(13)	9(17)	0.587
Preoperative CSFAβ42 (100pgml^–1^), mean ± SD	1.42 ± 0.43	3.01 ± 0.99	<0.001
Preoperative CSF Aβ40 (100pgml^–1^), mean ± SD	62.98 ± 12.33	26.45 ± 9.24	<0.001
Preoperative CSF Aβ42/Aβ40, mean ± SD	0.07 ± 0.03	0.13 ± 0.07	<0.001
Preoperative CSF T-tau (100pgml^–1^), mean ± SD	3.14 ± 2.06	1.19 ± 0.55	0.003
Preoperative CSF P-tau (pgml^–1^), mean ± SD	130.47 ± 0.51	69.02 ± 29.01	<0.001
Preoperative CSF PGRN (100pgml^–1^), mean ± SD	27.17 ± 8.73	30.49 ± 10.04	<0.001
Preoperative MMSE scores, median and 25–75 percentile	28(26–29)	28(27–29)	0.330
Duration of anesthesia (min), mean ± SD	133.97 ± 26.5	141.25 ± 30.1	0.963
Duration of surgery (min), mean ± SD	125.72 ± 25.13	129.47 ± 26.32	0.455
Intraoperative blood loss (ml), mean ± SD	582.44 ± 148.65	603.91 ± 152.77	0.465
Postoperative the highest MDAS (score), mean ± SD	22.75 ± 5.02	5.62 ± 2.43	<0.001
Postoperative the highest VAS(score), median and 25–75 percentile,	2(1–3)	2(1–3)	0.080
TICS-m (score), mean ± SD	36.2 ± 2.5	37.32 ± 3.5	0.079
WHOQOL-BREF score			
Physical domain(score), mean ± SD	68.3 ± 3.1	69.6 ± 3.9	0.068
Psychological domain (score), mean ± SD	75.1 ± 2.5	75.8 ± 2.9	0.193
Social relationships domain(score), mean ± SD	67.5 ± 2.9	67.3 ± 2.8	0.789
Environment domain (score), mean ± SD	83.6 ± 2.9	82.9 ± 2.5	0.205

*POD, postoperative delirium; MMSE, mini-mental state examination; ASA, American Society of Anesthesiologists; MDAS, memorial delirium assessment scale; VAS, Visual Analogue Scale/Score; SD, standard deviation; CSF, cerebrospinal fluid; Aβ_1–42_, amyloid-β_1–42_; Aβ_1–40_, amyloid-β_1–40_; T-tau, total tau; P-tau, phosphorylated tau; PGRN, Progranulin; TICS-m: Telephone Interview for Cognitive Status;WHOQOL-BREF: World Health Organization Quality of Life -brief version.*

### Risk Factors of Postoperative Delirium

In this study, the univariate logistic analysis showed that CSF PGRN, Aβ_42_/Aβ_40_, and CSF Aβ_42_ were protective factors of POD. CSF Aβ_40_, T-tau, and P-tau were the risk factors of POD.

After adjustment for age, sex, MMSE, educational level, and APOE ε4 carrier status, the multivariate logistic regression showed that CSF After adjustment for age, sex, MMSE, educational level, and APOE ε4 carrier status, the multivariate logistic regression showed that CSF PGRN, Aβ_42_/Aβ_40_, and Aβ_42_ were the independent protective factors of POD. However, CSF Aβ_40_, T-tau, and P-tau were still independent risk factors of POD (Table 2).

**TABLE 2:** Logistic analysis for Risk factors of POD patients adjusted by age, sex, MMSE, educational level and APOE ε4.

	Unadjusted		Adjusted	
	odds ratio (95% CI)	P-value	odds ratio (95% CI)	P-value
CSF PGRN (100pg • ml^–1^)	0.896(0.854–0.940)	0.001	0.073(0.646–0.832)	0.001
CSF Aβ_40_ (100pg • ml^–1^)	1.385(1.173–1.636)	0.001	1.781(1.161–2.731)	0.003
CSF Aβ_42_ (100pg • ml^–1^)	0.090(0.038–0.212)	0.001	0.082(0.033–0.203)	0.001
CSF Aβ_42/_Aβ_40_	0.000 (0.000–0.000)	0.001	0.000 (0.000–0.000)	0.001
CSF T-tau (100pg • ml^–1^)	3.438(1.915–6.170)	0.001	4.337(2.266–8.303)	0.001
CSF P-tau (pg • ml^–1^)	1.033(1.020–1.047)	0.001	1.046(1.029–1.064)	0.001

### Associations Between Cerebrospinal Fluid Progranulin and Cerebrospinal Fluid Biomarkers of Postoperative Delirium

**FIGURE 3:**
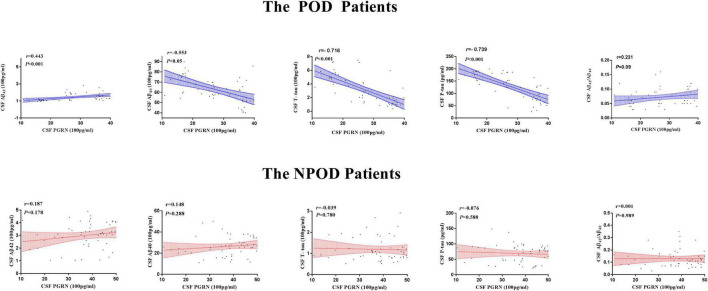
Associations of CSF PGRN and biomarkers of POD. Scatter plots represent the associations of CSF PGRN with CSF biomarkers (Aβ_42_, Aβ_40_, Aβ_42_/Aβ_40_, T-tau, and P-tau) of POD. The normalized regression coefficients (*r*) and *P*-values computed by multiple linear regression after adjustment for age, sex, education, and APOE ε4 carrier status are shown.

The associations between CSF PGThe associations between CSF PGRN and CSF biomarkers of POD were tested in linear regression models adjusted for age, sex, educational level, and APOE ε4 carrier status. As shown in Figure 3, CSF PGRN was positively associated with Aβ_42_ (*r* = 0.443, *P* < 0.001) and negatively associated with Aβ_40_ (*r* = − 0.553, *P* < 0.001), T-tau (*r* = − 0.716, *P* < 0.001), and P-tau (*r* = − 0.739, *P* < 0.001) in patients with POD. There were no significant associations of CSF PGRN with Aβ_42_/Aβ_40_ (*r* = 0.231, *P* = 0.09) in patients with POD. However, there were no significant associations of CSF PGRN with Aβ_42_ (*r* = 0187, *P* = 0.178), Aβ_40_ (*r* = 0.148, *P* = 0.288), Aβ_42_/Aβ_40_ (*r* = −0.001, *P* = 0.989), T-tau (*r* = − 0.039, *P* = 0.780), or P-tau (*r* = − 0.076, *P* = 0.588) in patients with NPOD. These findings indicated that higher CSF PGRN correlated with higher level of Aβ_42_, and lower levels of Aβ_40_ and tau pathology (Figure 3).

### The Predictive Effect of Progranulin on Postoperative Delirium

**FIGURE 4:**
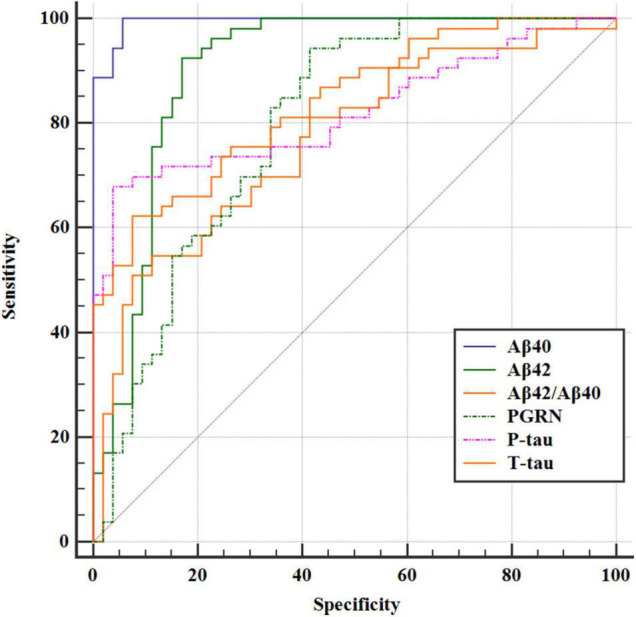
The ROC curve analysis of Aβ_42_, Aβ_40_, Aβ_42_/Aβ_40_, T-tau, P-tau, and PGRN showed that the concentrations of CSF PGRN had a high diagnostic value for POD.

Based on receiver operating characteristic (ROC) curve analysis, the AUC was 0.821 (95% CI = 0.735, 0.889) for P-tau between POD and NPOD groups. The AUC was 0.817 (95% CI = 0.730, 0.885) for T-tau between POD and NPOD groups. The AUC was 0.795 (95% CI = 0.706, 0.867) for PGRN between POD and NPOD groups. The AUC was 0.994 (95% CI = 0.956, 1.000) for Aβ_40_ between POD and NPOD groups. The AUC was 0.903 (95% CI = 0.830, 0.952) for Aβ_42_ between POD and NPOD groups. The AUC was 0.789 (95% CI = 0.699, 0.862) for Aβ_42_/Aβ_40_ between POD and NPOD groups. PGRN could predict POD occurrence among these study patients, suggesting that PGRN had a moderate predictive effect on POD occurrence (Figure 4).

### Causal Mediation Analyses

**FIGURE 5:**
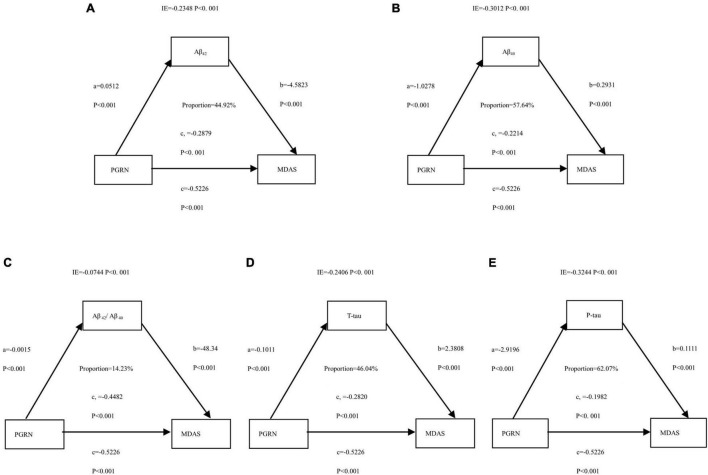
Mediation analyses with Memorial Delirium Assessment Scale (MDAS) as the cognitive outcome. In the PNDABLE (Perioperative Neurocognitive Disorder And Biomarker LifestylE study), the relationship between PGRN and MDAS was mediated by amyloid pathology indicated by **(A)** amyloid β 42 (Aβ_42_), **(B)** amyloid β 40 (Aβ_40_), **(C)** amyloid β 42/amyloid β 40 (Aβ_42_/Aβ_40_), **(D)** Total-tau (T-tau), and **(E)** phosphorylated total-tau (P-tau). IE, indirect effect.

The above findings suggested that CSF PGRN was not only a significant risk factor for POD but also a potential modulator of amyloid pathology. We then investigated whether CSF PGRN contributed to POD *via* modulating amyloid pathology. In the first regression, CSF PGRN was significantly associated with Aβ_42_, Aβ_40_, Aβ_42_/Aβ_40_, T-tau, and P-tau (*P* < 0.01). In the second equation, CSF PGRN showed a significant association with MDAS (*P* < 0.01). Finally, in the third equation, when the amyloid indicators and CSF PGRN were simultaneously included in the model, the influence of cognitive impairment remained significant. We found that the relationship between CSF PGRN and MDAS was mediated by amyloid pathology, including Aβ_42_, Aβ_40_, Aβ_42_/Aβ_40_, T-tau, and P-tau. The effect was considered partial mediation with the proportion of mediation varying from 14.23 to 62.07% (Figure 5).

## Discussion

This study was the first to reveal that CSF PGRN was associated with POD, and CSF PGRN was an independent protective factor for POD. Moreover, CSF Aβ_40_, T-tau, and P-tau were independent risk factors of POD. There were many risk factors for POD, such as advanced age, blood transfusion, anesthetic medications, and postoperative pain (Sugimoto et al., 2015). In recent years, researchers have tried to find ideal biological markers associated with the POD to reduce its occurrence. Aβ and Tau proteins, as biochemical indicators, are the main markers in the pathogenesis of POCD (Li et al., 2014). Some studies have also confirmed that preoperative CSF Aβ and Tau in patients with geriatrics are significantly correlated with the changes in postoperative cognitive function (Wu et al., 2018), consistent with this study.

In this study, the incidence of POD was 9.7%, which was consistent with the previous result of 3.6–41% (Leung et al., 2013). A previous study has shown that the incidence of POD after the total knee and hip replacement under spine anesthesia is 20% (Xie et al., 2014). Confusion assessment method is an internationally recognized method for evaluating the delirium status, with a sensitivity of 94–100% and a specificity of 90–95%. MDAS is a scoring system to rate delirium severity. It has a high degree of fit with MMSE which is commonly used in psychiatry to judge the severity of delirium (Breitbart et al., 1997). At present, MDAS has been widely used in clinical and research settings. Therefore, we used CAM and MDAS to evaluate the delirium status and the severity of delirium.

This study found that the influence of PGRN on cognition was partially mediated by amyloid pathology and tau protein in elderly adults. Therefore, it is reasonable to infer that amyloid pathology and tau protein could modulate the relationship of PGRN with cognition via mediation effects. PGRN is a multifunctional growth factor expressed in a variety of tissues and involved in many physiological and pathological processes (Arrant et al., 2020). It is widely expressed in various cells of the body. Some studies have found that PGRN is highly expressed in neurons and microglia cells. Little was previously known about the role of PGRN in the nervous system. However, since the discovery of PGRN genetic polymorphisms, the number of studies on the role of PGRN in the brain has increased rapidly. Studies have found that when PGRN is downregulated, it activates central cyclin-dependent kinase (CDK), which leads to reduced clearance of toxic Aβ42 in rat brain slices (Bennecib et al., 2000). Other studies have found that the content of PGRN in microglia cells around Aβ protein deposition increases in mice brain tissue (Hosokawa et al., 2018). Increased PGRN can activate microglial cells and stimulate them to engulf toxic Aβ around them, exerting neuroprotective effects in the animal model (Guan et al., 2020). In contrast, Aβ_42_ could downregulate PGRN expression in neurons and microglia through the alteration of signaling pathways. For example, PGRN expression is downregulated by toll-like receptor (TLR) ligands (e.g., LPS, the ligand for TLR4, and poly-IC, the ligand for TLR3) and proinflammatory cytokines (e.g., IL-1 and interferon-γ). Related pathways may be involved in downregulating PGRN expression in mouse models with AD at early disease stages. Progranulin expression could also be regulated by non-Aβ factors, including C-terminal hAPP fragments and their downstream signaling pathways. Moreover, PGRN could be cleaved by proteases (e.g., matrix metalloproteinases) into granulins that exert divergent biological functions (Suh et al., 2012). Therefore, we speculated that CSF PGRN might participate in Aβ protein deposition, thus leading to the occurrence of POD.

Neurofibrillary tangles are one of the main pathological features of neurodegenerative diseases, which are closely related to two major proteins—Tau protein and CDK (Goossens et al., 2018). Tau protein is found throughout the nervous system, and its hyperphosphorylation is one of the early cytoskeletal changes during the formation of nerve fiber tangle (NFT) in mouse models (Huang et al., 2018). Tau protein is modified by 2–3 phosphate groups. The phosphorylation and dephosphorylation of tau protein keep a dynamic balance, maintaining the stability of the cytoskeleton in mouse models (Zhang et al., 2018). In the pathological state of neurodegenerative diseases, tau protein has 9–10 phosphate groups, leading to its hyperphosphorylation and the formation of NFT (Simoes et al., 2020). Hyperphosphorylated tau protein loses its original functions and cannot promote microtubule focusing and maintain cytoskeleton stability (Monteiro-Fernandes et al., 2021). Neurofibrillary tangles and neuronal loss caused by tau hyperphosphorylation lead to cognitive dysfunction in mouse models (Fan et al., 2020). In this study, we explored the associations between CSF PGRN and CSF biomarkers for POD to further provide a theoretical basis for early warning and intervention of POD. Our results showed that CSF PGRN was positively correlated with Aβ_42_ and negatively correlated with Aβ_40_, T-tau, and P-tau. In NPOD subjects, the significant associations of PGRN with Aβ_42_, Aβ_40_, T-tau, and P-tau disappeared. These findings suggested that CSF PGRN might indeed be associated with POD. Therefore, we speculated that CSF PGRN might participate in Aβ protein deposition and Tau protein phosphorylation, thus leading to the occurrence of POD.

Evered et al. found in their studies of cardiac surgery and non-cardiac surgery that preoperative low Aβ levels were associated with the occurrence of POD (Evered et al., 2009). In another study, the basal level of Aβ_1–40_ was higher in patients with geriatrics, suggesting that Aβ_1–40_ might be one of the reasons for the higher incidence of POCD in the elderly. Tau protein is a microtubule-associated protein widely expressed in the nervous system. It binds to and stabilizes the microtubule system and performs various functions by interacting with a variety of proteins. Hyperphosphorylated tau protein can cause impaired neurotransmitters and a large number of neuronal tangles in the brain, leading to impaired memory, learning, and cognitive function. Planel et al. found that after administration of isoflurane anesthesia in mice, tau protein was hyperphosphorylated in the hippocampus, leading to decreased cognitive function in a mouse model (Planel et al., 2009). Decreased CSF PGRN and its effects have been observed in the brains of patients with neurodegenerative diseases. Decreased expression of PGRN in microglia cells around amyloid plaques is a self-protective mechanism to prevent cell damage, which offers prospects for the application of CSF PGRN as a biomarker for patients with cognitive dysfunction.

The ROC curve analysis of Aβ_42_, Aβ_40_, Aβ_42_/Aβ_40_, T-tau, P-tau, and PGRN concentrations in CSF showed that PGRN had the reasonably good diagnostic value [AUC was 0.795 (95% CI = 0.706, 0.867)]. At the same time, this study shows that Aβ_42_, Aβ_40_, Aβ_42_/Aβ_40_, T-tau, P-tau, and PGRN have a good correlation for patients with POD. Therefore, our results show that PGRN concentrations in preoperative CSF can predict the occurrence and development of POD. It is the future direction of our research to prove the findings in animal experiments and explore the relevant mechanisms.

Our investigation had three limitations. First, our findings should be replicated in subjects with longitudinal data in the future. Second, CSF collection is an invasive procedure. Monitoring the concentration of PGRN in the plasma of patients will make the clinical examination more convenient. This study will monitor the progression of the disease by measuring the changes in PGRN concentration in the peripheral blood in the future. Third, it is the future direction of our research to prove our findings in animal experiments and explore the underlying mechanisms.

## Conclusion

Cerebrospinal fluid PGRN may be a reasonably good prognostic factor for POD development. Overall, amyloid pathology and tau protein might partially mediate the influence of PGRN on POD.

## Data Availability Statement

The raw data supporting the conclusions of this article will be made available by the authors, without undue reservation.

## Ethics Statement

The studies involving human participants were reviewed and approved by the Ethics Committee of Qingdao Municipal Hospital. The patients/participants provided their written informed consent to participate in this study. Written informed consent was obtained from the individual(s) for the publication of any potentially identifiable images or data included in this article.

## Author Contributions

YB conceived this study. JW, YL, XD, and FL performed the experiments. RD, XL, XS, and BW analyzed the data. XL, RD, and BW performed the experiments and wrote and revised this manuscript. All authors have contributed to the manuscript revising and editing critically for important intellectual content, given final approval of the version, agreed to be accountable for all aspects of the work presented in this study, and read and approved this manuscript.

## Conflict of Interest

The authors declare that the research was conducted in the absence of any commercial or financial relationships that could be construed as a potential conflict of interest.

## Publisher’s Note

All claims expressed in this article are solely those of the authors and do not necessarily represent those of their affiliated organizations, or those of the publisher, the editors and the reviewers. Any product that may be evaluated in this article, or claim that may be made by its manufacturer, is not guaranteed or endorsed by the publisher.

## Abbreviations

PGRN: progranulin
AD: Alzheimer’s disease
POD: postoperative delirium
NPOD: no postoperative delirium
CSF: cerebrospinal fluid
ROC: receiver operating characteristic curve
CDK: cyclin-dependent kinase
NFT: nerve fiber tangles
A β: β-amyloid
CAM: Confusion Assessment Method
MDAS: Modified Dental Anxiety Scale
ELISA: enzyme linked immunosorbent assay
MMSE: Mini-mental State Examination
PACU: post-anesthesia care unit
ASA: American Society of Anesthesiologist.
